# Lumbar Plexopathy and Exploration Following an Oblique Lumbar Interbody Fusion (OLIF) iFactor‐Associated Complication: A Case Report

**DOI:** 10.1155/cro/1328979

**Published:** 2026-05-20

**Authors:** Charles Taylor, Arka Banerjee, Hasan Raza, James Geddes, Liam Rose, Margot Montgommery, Mohammed Abdelhamid, Timothy Bishop, Jason Bernard, Darren Lui

**Affiliations:** ^1^ St George′s University of London St George′s Library, London, UK; ^2^ Department of Orthopaedics, St George′s NHS Foundation Trust, London, UK; ^3^ Vascular Surgery Department, St. George′s NHS Foundation Trust, London, UK

**Keywords:** case report, iFactor, lumbar plexopathy, OLIF

## Abstract

**Background:**

Oblique lumbar interbody fusion (OLIF) is a minimally invasive surgical technique used for lumbar spine stabilisation, known for reducing the morbidity associated with traditional posterior lumbar fusion techniques. iFactor, a synthetic bone graft composed of P‐15 osteogenic cell‐binding peptide and anorganic bone mineral (ABM), is often used in such procedures for its reported high fusion rates and minimal complications. iFactor reports to only form bone when grafted into bone tissue, and not soft tissue. This case report presents a rare but significant complication requiring reoperation due to extruded iFactor putty.

**Case Presentation:**

A 65‐year‐old female smoker with a history of failed conservative management for back and right radicular leg pain underwent an OLIF procedure at the L4/5 intervertebral level. Postoperatively, she was well for the first 2 weeks but suddenly developed left leg and lower back pain. Delayed diagnosis of a rare complication was detected only after postoperative CT scans were analysed 6 months after surgery. Initial imaging suggested exogenous bone growth affecting the lumbar plexus.

**Management and Outcome:**

Diagnostic nerve blocks revealed that L3 and L4 roots were affected. Exploratory surgery importantly revealed that de novo bone had not encased the nerve roots but revealed a chalky paste appearance consistent with extruded iFactor putty. Following the surgery, the patient′s symptoms improved considerably, emphasising the importance of accurate diagnosis and multidisciplinary team (MDT) involvement.

**Conclusion:**

This is a rare complication following OLIF, allowing extruded artificial graft to encase part of the lumbar plexus. This case supports the manufacturer′s assertion that iFactor does not lead to exogenous bone growth. The surgical exploration revealed a chalk‐like paste that was irritating the nerve plexus and highlights that surgical debridement of this substance under the psoas muscle is possible. This case also highlights that exploration of extruded bone grafts can be considered for patients presenting similarly after spinal fusion surgeries involving bone grafts.

## 1. Introduction

There are numerous surgical approaches described in the literature for lumbar interbody fusion. These include: posterior, transforaminal, minimally invasive transforaminal, oblique, lateral and anterior. However, the optimal approach remains a topic for debate [1, 2]. Oblique approach surgery may be considered an excellent technique due to its minimally invasive nature, shorter hospital stays and reduced rates of iatrogenic complications [3].

Oblique lumbar interbody fusion (OLIF) is a minimally invasive technique for indirect decompression of neural elements [3, 4]. During the OLIF procedure, the surgeon uses a retroperitoneal anterior‐to‐psoas approach, which minimizes muscle disruption compared with traditional open surgeries [3, 5]. OLIF is known for its potential benefits, including less postoperative pain, shorter recovery time and reduced risk of complications compared with more invasive techniques [3]. This approach limits the muscle injury–associated posterior spinal surgery such as posterior lumbar interbody fusion (PLIF) and transforaminal lumbar interbody fusion (TLIF) [6]. There are some advantages to the OLIF technique over posterior‐based approaches. Firstly, the size of the interbody cage has a larger surface area allowing apophyseal support limiting subsidence. Larger volumes of bone graft can be inserted into these cages. There is a superior ability to control lordosis with respect to sagittal balance matching lumbar lordosis with pelvic incidence. It has a good safety profile [7] in complex spinal surgery helping to obviate the use of pedicle subtraction osteotomy.

Interbody spinal fusions can use a variety of bone grafts ranging from autologous bone to synthetic. iFactor bone graft products are commonly used to replace or augment the use of autologous bone grafts. iFactor is a biologic bone graft made of P‐15 osteogenic cell‐binding peptide bound to an anorganic bone mineral (ABM) [8]. The use of iFactor has been shown to be superior to autologous bone and BNP (B‐type natriuretic peptide) in PLIF [6] and ALIF [7] and is supported by several clinical studies, including the IDE [8] and IVANOS [9] studies, which show a 2.5% greater fusion rate with minimal complications.

iFactor is based on the biological activity of P‐15 which is responsible for the attachment and proliferation of osteogenic cells [8, 10]. P‐15 is immobilised on the ABM substrate and enhances cell binding and consequentially speeds the process of new bone formation only at the implant site. As it is surface‐bound, any cellular activity from P‐15 osteogenic cell‐binding peptide is limited to the implant surface, and therefore, theoretically ectopic bone growth is not possible [7, 10].

In the present case, we describe a 65‐year‐old female life‐long smoker who underwent an OLIF procedure and, after an uncomplicated surgery, developed an acute left leg sciatica 2 weeks after surgery. CT scans revealed suspected exogenous bone encasing the left lumbar plexus. Despite exhaustive conservative management and several MDT discussions, which involved controversy amongst several experts at complex spine MDTs, exploratory surgery was conducted.

## 2. Case Presentation

We present the case of a 65‐year‐old female life‐long smoker who is normally independent. She presented with a history of recurrent acute‐on‐chronic back pain over 18 months. These recurrent episodes followed a similar pattern: the patient would go to bed pain free and in the morning would experience extreme lower back pain that severely limited her mobility, taking her 15 min to get out of bed.

Initially, the sudden onset episodes lasted for 2 weeks, with severe restriction in movement secondary to pain that gradually improved. However, more recent episodes required 3–5 weeks before complete resolution of lower back pain and return to normal functional status.

The lower back pain was accompanied by paraesthesia in the posterior aspects of her back, legs and calves, with occasional radiation of paraesthesia into her feet. She also experienced sciatica‐type pain down her right lower limbs leading to an antalgic gait. During these episodes, she displayed a sort of Gowan′s sign when getting out of a chair. She denied any saddle paraesthesia or bowel/bladder disturbances during these episodes.

The clinical exam revealed that there was discogenic pain on forward flexion felt as a band of pain at the level of the iliac crest consistent with L4/5 pain. There was pain in the L4/5 facet joints on extension, worse with left and right extension.

Her past medical history was significant for a previous spinal fracture which caused chronic neck pain and a Morton′s neuroma; however, she describes the paraesthesia and sciatica‐type pain associated with the lower back pain to significantly differ from what she experiences with the Morton′s neuroma. Paracetamol and co‐codamol were of little analgesic effect.

**Figure 1:**
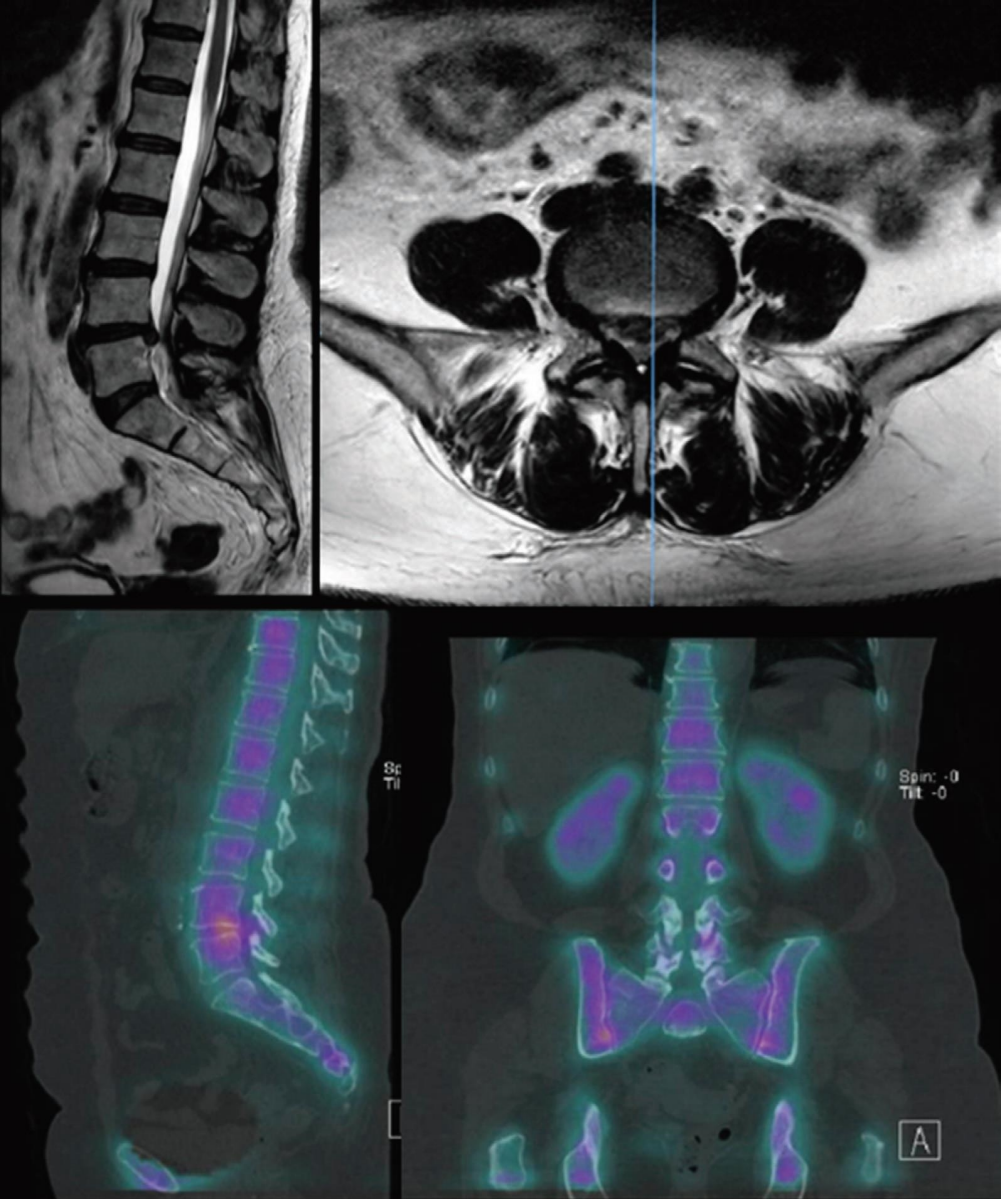
Preoperative MRI.

The pain was initially managed with a caudal epidural in September 2021, and finally planned for a two‐stage OLIF in November 2021. Preoperative MRI showed posterior protrusion of L4/5 intervertebral disc with impingement on the neural contents of the spinal canal (Figure 1).

### 2.1. Initial Operation

The posterior protrusion of the L4/5 intervertebral disc with impingement on the neural contents of the spinal canal identified on MRI (Figure 1) was treated with a retroperitoneal OLIF via a left anterior‐to‐psoas approach. This is where the psoas is reflected off the interbody discs, creating a potential space.

**Figure 2:**
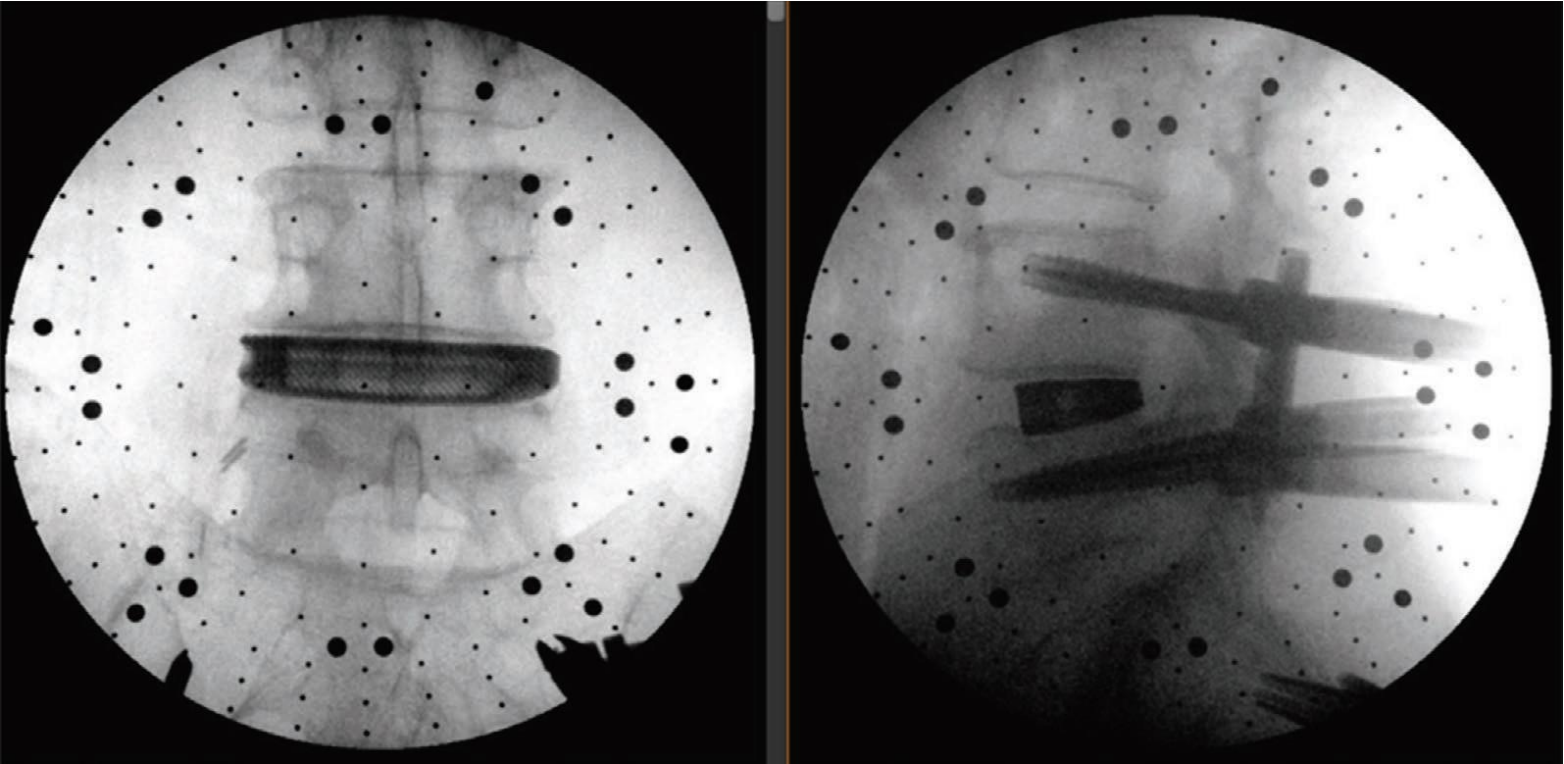
Intraoperative X‐ray imaging.

**Figure 3:**
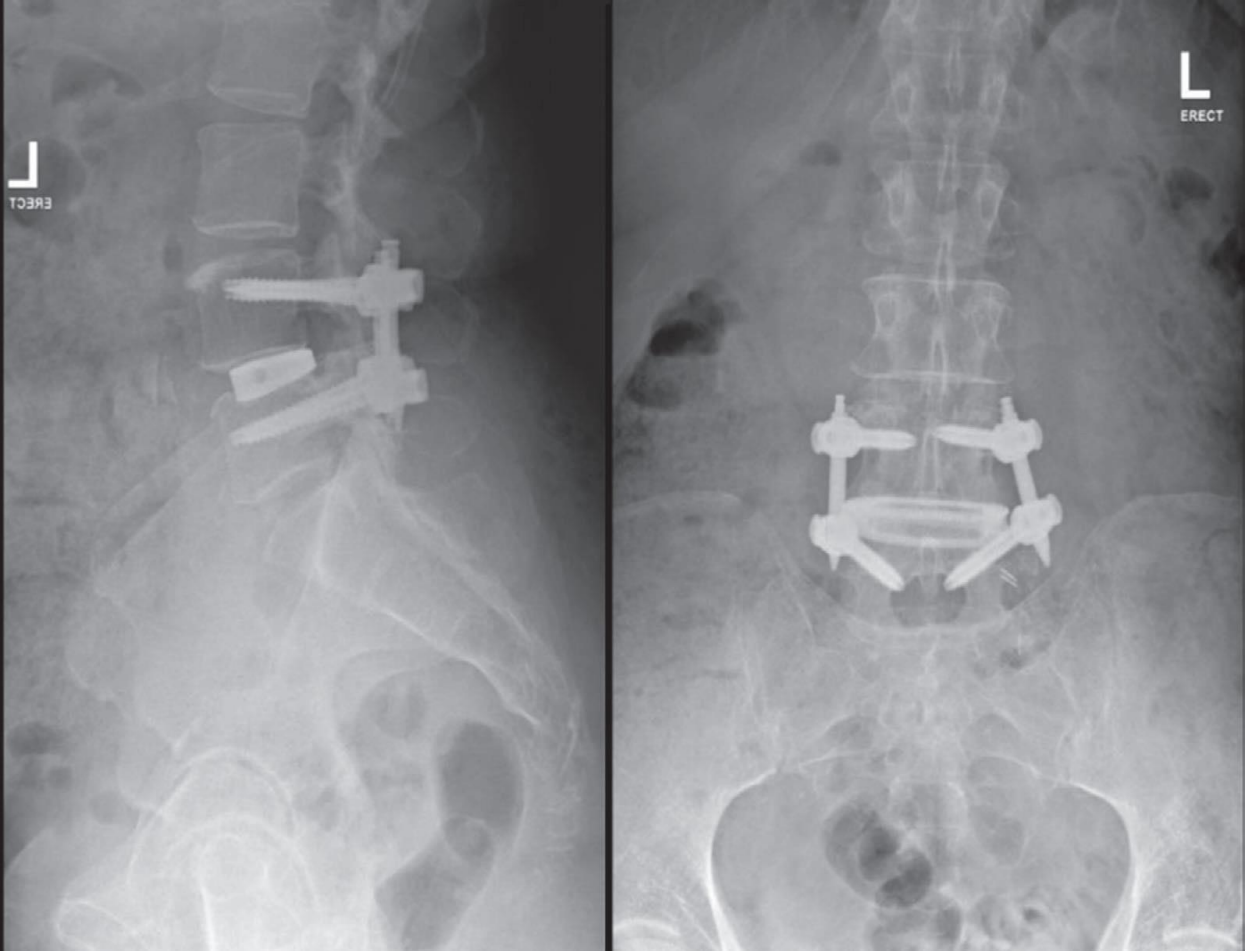
Postoperative X‐ray.

iFactor putty was injected into the cage prior to insertion of the cage into the L4/5 intervertebral space. There were no complications during the procedure, and intraoperative X‐ray imaging identified appropriate positioning of the cage in the L4/5 intervertebral space after the discectomy (Figure 2). The psoas muscle was left to fall back onto the disc space. Postoperative x‐rays were satisfactory (Figure 3) with no significant clinical complications. On Day 1, she mobilised and walked, and by Day 4, she was able to walk the stairs independently and was fit for discharge.

### 2.2. Follow‐Up

Postoperatively, she recovered well. However, 2 weeks postoperatively, in December 2021, she developed acute left leg pain, in contrast to her preoperative complaint of right leg pain.

The patient was initially managed conservatively. Subsequently, she underwent a series of diagnostic injections of limited efficacy. She underwent nerve blocks and epidurals including: CT‐guided left root block in January 2022 with minimal effect; caudal epidural and facet blocks in March 2022 with moderate effect; radiofrequency ablation of L5/S1 facet joints in May 2022 with minimal effect; left L4 block and bilateral L5/S1 facet joint injections in September 2022 with good effect; left L4 block in December 2022 with minimal effect; and finally, a left L3, L4, L5 nerve root block in March 2023 with excellent pain relief but only for 1 week. It must be noted that the initial CT in January 2022 did not identify any lumbar plexus changes.

**Figure 4:**
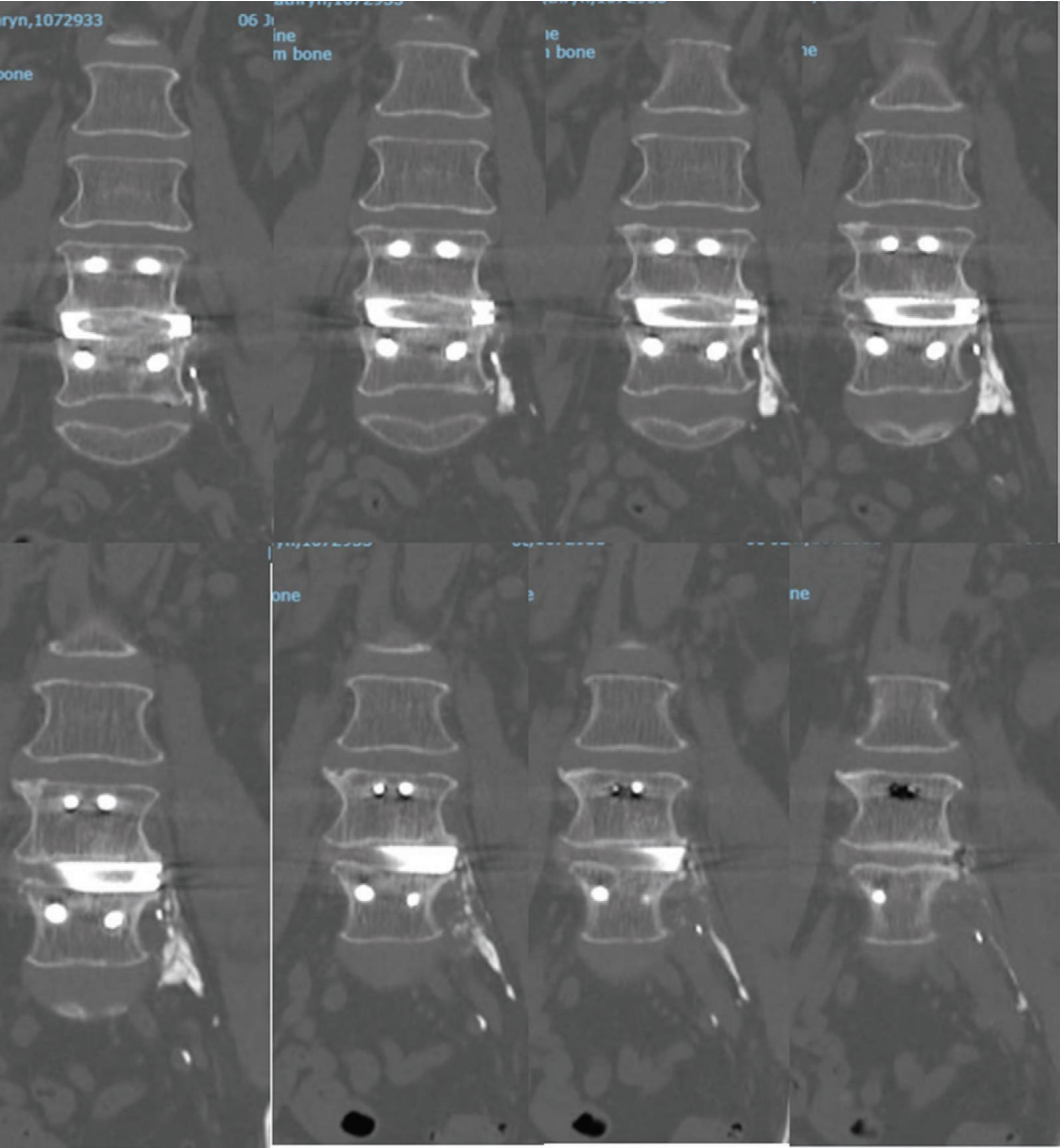
Coronal CT displaying extruded iFactor putty.

The failure of conservative treatment and interventional procedures prompted rediscussion in the multidisciplinary team meeting in July 2022, where coronal CT images from January 2022 were reviewed. This review identified what appeared to be exogenous bone growth affecting the lumbar plexus (Figure 4). This was thought to be triggering the lower back pain and left leg sciatica‐type radiation.

The 6‐month delay led to the dilemma of postulating whether the imaging showed exogenous bone growth from the iFactor bone graft that extruded from the cage into the sub‐psoas space. The device manufacturer was contacted about this, and they reported that the iFactor should not cause bone growth outside the interbody space in the cage. This postoperative presentation was initially thought to be irritation from the P‐15 osteogenic cell‐binding peptide [8] in the putty.

Consent for the exploratory procedure was cautionary. The decision to explore was not unanimous, and indeed controversial. Many surgeons felt it would be inappropriate to explore given that the CT scan depicted what seemed to be solid bone encasing the lumbar plexus, and that any operation could cause more harm or be futile at best. The patient was counselled over several appointments and decided that she would like to trial exploratory surgery.

**Figure 5:**
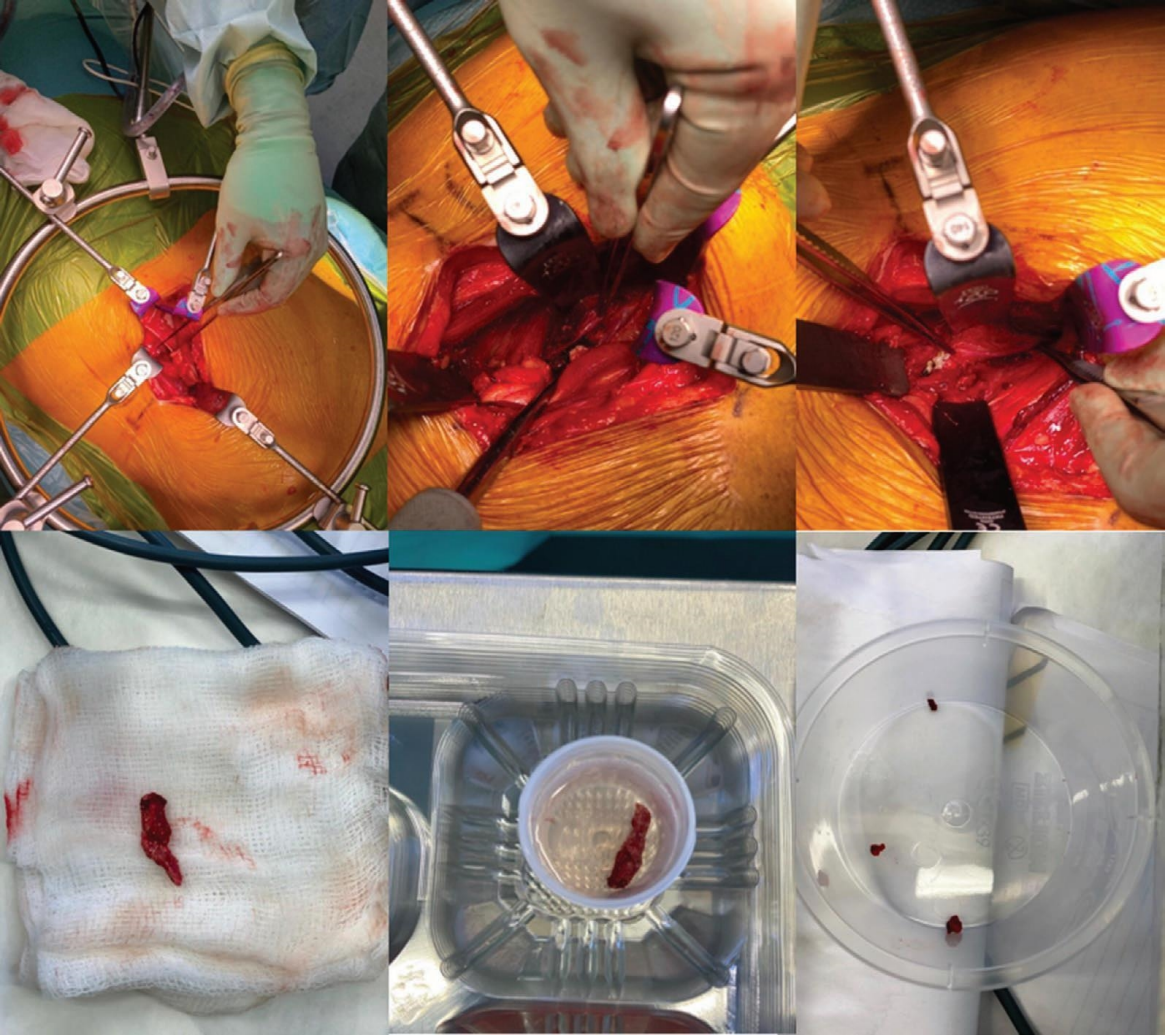
Lumbar exploration and removal of putty.

A vascular surgeon was consulted with regards to the surgical approach to ensure minimum risk to surrounding vascular structures. The patient therefore underwent exploratory surgery. On direct visualisation during the exploratory surgery, the ‘exogenous bone growth’ identified on CT was identified to be a chalky paste and not exogenous bone in support of the device manufacturer′s claims (Figure 5). There was no evidence of solid ectopic bone formation. Instead, granular, nonossified material consistent with extruded iFactor putty was identified along the psoas–spine interface. A tubular segment of this material, approximately 3–4 cm in length, was found adherent to a lumbar nerve root and was carefully dissected and removed. This material did not resemble mature cortical or trabecular bone.

The chalk‐like substance was manually removed methodically, as best as possible under direct vision. Copious washout was utilised to ensure maximal removal of the particles. Visually, not all particles could be removed, particularly the caudal parts of the plexus, which remained technically challenging to visualise and remove safely. Additionally, at re‐exploration, good fusion was observed, and L4/5 and quality of fusion was not a concern. Therefore, following direct visualisation, the primary mechanism underlying the patient′s symptoms was considered mechanical extrusion rather than a biological failure of fusion or exogenous ossification. After the revision procedure, the patient demonstrated significant improvement in quality of life and functional outcomes, with an EQ‐5D score of 78 (Baseline 10) and an Oswestry Disability Index (ODI) of 42 (Baseline 67). At 2‐year follow‐up, improvements were sustained, with an EQ‐5D score of 70 and ODI of 38. Pain Catastrophizing Scale (PCS) scores remained below the threshold for clinical significance at both 1 (27) and 2 years (21), indicating no significant maladaptive pain‐related psychological burden. These findings suggest durable clinical benefit following surgical removal of the extruded graft material.

## 3. Discussion

The case presented highlights a rare but significant complication following an OLIF procedure using the iFactor bone graft putty. Despite technical advantages, this case underscores the potential complications associated with the use of any bone graft in an anterior‐to‐psoas approach, necessitating further exploration and intervention. This complication may have arisen due to the potential space created by reflecting the left psoas off the disc space, allowing for a space for extrusion of bone graft.

The delayed onset of symptoms at 2 weeks suggests a possible multifactorial mechanism. A primary explanation is progressive mechanical extrusion of graft. Early postoperative mobilisation, spinal loading and psoas muscle contraction may have contributed to gradual displacement of iFactor from the interbody cage. The absence of immediate symptoms supports a threshold‐dependent effect, whereby gradual accumulation of extruded material eventually resulted in sufficient mechanical irritation of the lumbar plexus to produce radiculopathy. A biological contribution may also be relevant. Although P‐15 grafts are designed for site‐specific osteogenesis, extruded material in soft tissue may provoke a low‐grade inflammatory response. Alternative causes such as postoperative haematoma or nerve oedema are less likely given the intraoperative findings but cannot be totally excluded.

This case supports the manufacturer′s claim that iFactor does not cause exogenous bone growth. Initially, the patient′s new onset of pain was misinterpreted as such, contradicting the theoretical properties of iFactor. Detailed imaging review and surgical exploration confirmed that the complication was due to extruded putty rather than bone growth, validating the manufacturer′s assertion that P‐15 osteogenic cell‐binding peptide and ABM promote bone formation at the site of implantation without the risk of ectopic growth.

The management of this complication involved conservative measures and interventional procedures, including nerve blocks and epidural injections. Despite these interventions, the patient′s persistent pain led to the decision for exploratory surgery. Due to persistent pain, one should have a high degree of suspicion and utilise appropriate imaging. Furthermore, one should examine multiplanar reconstructions of imaging—sagittal, coronal and axial—to look for clues that may cause mechanical irritation of nerves. Intraoperatively, there was a chalky particulate paste of extruded iFactor putty encasing the lumbar plexus, which was identified as the source of the mechanical nerve irritation.

This case highlights that the CT scan can be deceiving, where similar lesions may be erroneously reported as exogenous solid bone. Whereas the reality is that any form of calcium can resemble solid bone, even if it is in fact particulate in nature.

Intraoperative findings confirmed that the material surrounding the lumbar plexus represented extruded iFactor putty rather than exogenous bone. However, the patient′s smoking status may have contributed to the extruded graft not creating bone, as smoking is a well‐recognised risk factor for impaired bone formation and healing. However, this is thought to be unlikely given that iFactor, unlike growth factor products, only stimulates bone growth in the presence of osteoblasts and extrusion into the sub‐psoas space would likely prevent bone formation irrespective of smoking status.

Finally, in this case, a 10 × 50 × 22‐mm, 12° Stryker Cascadia Lateral 3D interbody cage was inserted with approximately 5 cc of iFactor graft. Fixation was achieved with screws in a direct lateral trajectory. Notably, this cage design is typically utilised in lateral (LLIF/XLIF) approaches and is characterised by its long footprint spanning the apophyseal ring, which is advantageous in reducing subsidence due to load distribution across stronger cortical bone. However, the use of a longer LLIF‐type cage within an oblique approach may have implications for a relatively more posterior cage position compared with a standard OLIF‐specific cage. Here, the slightly posterior positioning may have reduced the margin between the cage and adjacent neural structures, including the lumbar plexus. Although it is unlikely that this positioning is facilitating extrusion of graft material. In the event of extrusion, it may increase the risk of neural structure involvement.

This case underscores several key learning points. This may be the first description in literature of bone graft extruding postoperatively in a delayed fashion. The OLIF technique utilises a left‐sided anterior‐to‐psoas approach which may create a potential space for extrusion of bone graft. The delayed sudden left‐sided radiculopathy should cause consideration for early CT scan to identify atypical pathology. There should also be a degree of suspicion regarding any atypical calcific lesions seen around the bone graft on CT, as the lesion may represent extruded graft that can be safely removed (to an extent) and not exogenous bone growth. Despite the safe profile of the OLIF approach, atypical complications can occur. Surgeons should continue to remain vigilant for atypical presentations and complications following spinal fusion procedures.

## 4. Conclusion

This case report underscores the importance of accurate diagnosis and the role of multidisciplinary management in resolving postoperative complications. Despite the safety profile of the OLIF approach, atypical complications can lead to significant patient morbidity. On imaging, graft material may radiologically mimic bone growth, although manufacturer specifications on the risk of ectopic bone growth should be considered. This case illustrates how extrusion of graft material can clinically and radiologically mimic bone growth. The findings support the manufacturer′s claim that exogenous bone growth is not currently possible with iFactor. Future considerations should include thorough preoperative planning and postoperative monitoring to identify and address potential complications early.

## 5. Limitations

This report is somewhat limited by the absence of a detailed quantitative assessment of cage positioning and formal analysis of potential contributing factors such as distance from posterior vertebral body line and sagittal position ratio. Such evaluation could provide additional insights into technical factors influencing cage placement and could inform optimisation of surgical technique in future cases. However, the intraoperative findings and subsequent symptom resolution following removal of extruded graft material strongly support that the patient′s symptoms were due to mechanical irritation from extruded iFactor, rather than cage position. Although cage positioning may be a contributing factor to direction of graft extrusion, it is unlikely to represent the underlying aetiology.

## Funding

No funding was received for this manuscript.

## Conflicts of Interest

The authors declare no conflicts of interest.

## Data Availability

The data that support the findings of this study are available from the corresponding author upon reasonable request
